# Sixth International Symposium on Recent Advances in Environmental Health Research

**DOI:** 10.3390/ijerph7052131

**Published:** 2010-05-04

**Authors:** Paul B. Tchounwou

**Affiliations:** National Institutes of Health RCMI, Center for Environmental Health, College of Science, Engineering and Technology, Jackson State University, 1400 Lynch Street, Box 18750, Jackson, MS 39217, USA; E-Mail: paul.b.tchounwou@jsums.edu

This special issue of *International Journal of Environmental Research and Public Health* highlights selected papers presented at the *Sixth International Symposium on Recent Advances in Environmental Health Research* organized by Jackson State University (JSU) from September 13−16, 2009 at the Marriott Hotel in Jackson, Mississippi, USA. The Symposium was built upon the overwhelming success of previous symposia hosted by JSU and co-sponsored by the National Institutes of Health (NIH) RCMI-Center for Environmental Health, the U.S. Department of Education Title III Graduate Education Program, the U.S. Environmental Protection Agency, the JSU Office of Academic Affairs, and the JSU Office of Research and Federal Relations.

In maintaining the strong tradition initiated with the first symposium in 2004, the program of the *Sixth International Symposium on Recent Advances in Environmental Health Research* provided a strong forum to contribute global solutions to major environmental and human health challenges. The Symposium had a special appeal to international scientists who have been committed to bioenvironmental and public health research, studying the toxic mechanisms of action of various environmental agents, developing new approaches for detecting or remedying environmental damage, identifying and characterizing genes involved in the manifestation of environmentally-related diseases, conducting basic and translational research, and providing the public and policy makers with scientific tools that are critical for environmental and human health decision-making. This integrated approach offers practical and cost-effective approaches to improving environmental quality and public health though translation of new scientific knowledge and discoveries from fundamental and basic sciences to environmental sustainability and public health protection. The symposium also offered unparalleled opportunities for networking and exchange of ideas, leading to scientific collaborations, resources sharing, and strategic planning for multi- and inter-disciplinary approaches to environmental and public health research.

Building on the foundation of the first, second, third, fourth and fifth symposia, the *Sixth International Symposium on Recent Advances in Environmental Health Research* served as a platform to exchange innovative ideas and communicate the latest advances in scientific research and new developments on important environmental and human health topics, including the following:

- ***Nanoscience, Nanotechnology and Nanotoxicology:*** In recent years, nanoscience and nanotechnology have gained a great deal of public interest due to the needs and applications of nanomaterials in almost all areas of human endeavors including industry, agriculture, business, medicine and public health. Hence, nanoscience and nanotechnology rank among the most prominent and rapidly emerging fields that have provided opportunities to individuals with various academic backgrounds (chemists, biologists, physicist, material scientists, engineers, medical specialists and toxicologists) and scientific expertise to understand things on the nanoscale. While there have been significant advances in nanoscience and nanotechnology, there have been concerns that the wide production and utilization of nanomaterials is rapidly overtaking efforts to evaluate their toxicity to humans and the environment. To date, very few studies have focused on the evaluation of the impact of nanomaterials on human health. Limited toxicological data indicate that nanomaterials exposure poses a potential risk to biological systems. Symposium presentations focused on the toxicological and health effects of nanomaterials including single and multiwalled carbon nanotudes, and quantum dots, as well as on the application of nanomaterials in biotechnology.
- ***New Frontiers in Environmental Health Research:*** The causes of most human diseases have been attributed to the complex interactions between genetic factors and environmental exposures. Hence, control and prevention measures highly rely on the understanding of the cause and effect relationships between these factors and disease development. In recent years, new areas of research such as toxicogenomics, proteomics, and functional genomics have emerged, with the aim of understanding molecular mechanisms of health and disease. Also, the recent advances in the molecular biology of the cell cycle regulation have given new life to our understanding of cancer in particular, and the idea that defects of regulation in cancer cells may partially explain successes that have been achieved in cancer chemotherapy. Specific areas of symposium research presentations included gene expression studies, proteomics, gene-environment interactions, functional genomics, biomarkers of effect, sensitivity and effect, signal transduction and gene activation; and molecular targets of disease chemotherapy.
- ***Environmental Toxicology and Health Risk Assessment:*** Growing public awareness of the potential risk to humans from toxic chemicals in the environment has generated demand for new and improved methods for toxicity assessment and rational means for estimating health risk. Many environmental agents such as metal ions, polycyclic aromatic hydrocarbons, pesticides/herbicides, UV-light, food additives, and viruses are known to induce various types of illnesses including cancer in humans. Several symposium presentations dealt with research elucidating the cellular and molecular mechanisms by which these environmental agents induce toxicity, mutagenesis, and carcinogenesis, as well as research on hazard assessment of exposure to physical, chemical and biological agents; dose-response evaluation and model development; exposure assessment analysis; and health risk characterization; and management.
- ***Emerging Topics in Computational Biology, and Environmental Modeling:*** Using of computational methods and procedures to investigate environmental and biological phenomena has made remarkable progresses. This field includes analysis of human genome data, prediction of DNA and protein structure and function, design of biomaterials and therapeutic agents, studies into small molecule-biomacromolecule interactions, and other related computational methods development. Therefore, several symposium presentations dealt with the computational analysis of the physical and chemical properties of several environmental compounds, as well as on quantitative structure activity relationship (QSAR) studies for developing predictive toxicology models associated with exposure to these compounds.
- ***Health Disparities and Environmental Security:*** In recent years health disparities and biological and chemical terrorism have emerged as major issues in public safety and homeland security. With recent advances in laboratory technologies, it is often possible to measure specific genetic variations as risk factors for specific types of disease. Equally important is the evaluation of the role of modifier factors such as environmental exposures or other genes that may exacerbate the genetic risk leading to differences in disease susceptibility among individuals. Since the events of September 11, 2001 regarding the attacks on the World Trade Center and the Pentagon, and the subsequent anthrax attacks on several people, our collective thinking with regard to our vulnerability to terrorism has completely changed. The specific areas of research presentations included the following: health disparities and cancer; health disparities and heart disease; health disparities and infectious diseases; and bioterrorism/chemical terrorism.
- ***Medical Geology and Human Health:*** Recent concerns over health-related issues arising from exposure to environmental substances have raised substantial interest in a new field termed “medical geology”. In fact, naturally occurring toxic metals such as arsenic, cadmium, lead, and mercury are now known to cause serious public health problems in several areas of the world. Likewise, the geographical distributions of several infectious diseases such as malaria, meningitis, and schistosomiasis, have been linked to intrinsic climatic and environmental factors. Research on this topic dealt with disease ecology, toxicology, pathology and/or epidemiology with regard to the emerging subject of medical geology.
- ***Natural Resources Damage Assessment and Management:*** Several environmental influences including natural and anthropogenic factors have been linked to ecosystem vulnerability. Monitoring and assessment data are therefore needed for science-based decision-making with regard to environmental management. Papers for presentation on this topic included those related to: a) conceptual modeling for ecological risk assessment, b) assessment of the physical, chemical, and biological characteristics of specific ecosystems, c) applications of GIS and remote sensing technology to environmental assessment and management, and d) bioindicators for environmental management.

The symposium attracted 302 participants from 22 countries representing all five continents. A total of 194 scientific presentations were made across the disciplines of environmental health, biomedical and clinical sciences, and public health. As listed above, the scientific program was composed of seven plenary sessions where oral/platform presentations were given by more than 40 invited speakers. In addition, there were two poster sessions—one for faculty and professional scientists, and one for students that included awards for best posters presentations at four levels of the educational pipeline including high school, undergraduate, master’s and doctorate levels. The submitted full length manuscripts were peer-reviewed, and selected for publication by experts in their respective fields. The peer-review process was conducted as illustrated in Figure 1.

I wish to extend special thanks to Dr. Brian Athey (Founding Associate Director of the Center for Computational Medicine and Bioinformatics, Chair of Department of Computational Medicine and Bioinformatics, and Director of Academic Informatics and Information Technology at the University of Michigan School of Medicine in Ann Arbor, MI), and Dr. Bailus Walker (Professor of Environmental and Occupational Health at Howard University College of Medicine in Washington, DC) for serving as Distinguished Speakers for the Biomedical Sciences and Health Information Lecture Series that is held in conjunction with the Symposium. Dr. Athey spoke on behalf of Dr. Gilbert Omenn (Director of the Center for Computational Medicine and Bioinformatics at the University of Michigan) on the *Strategies for Proteomic Profiling of Cancer Specimens and Plasma in Mouse Models of Human Cancers*, and Dr. Walker spoke on the *Influence of Environmental Factors on Health Disparities*. Other platform presentations and keynote addresses were made by prominent biomedical and environmental health scientists with research expertise in cancer, diabetes, HIV/AIDS, infectious and parasitic diseases, cardiovascular diseases, neurodegenerative diseases, gene-environment interactions, nanoscience and nanomedicine, emerging technologies, health disparities and other environmentally-related illnesses. These important health issues were associated with the symposium topics.

Special thanks are extended to Mrs. Rose Foster and Mrs. Wilma Templin-Branner of Oak Ridge Institute for Science and Education, Dr. Kenneth Ndebele and Dr. Barbara Graham for their continued support and help with the organization of the pre-symposium workshop on the *National Library of Medicine Web-Based Resources for Environmental Health and Biomedical Research.* Major emphasis of the workshop was training participants on how to access and retrieve important environmental health and biomedical research information from the Toxicology Network database and other relevant web-based biomedical resources. Special thanks are also extended Dr. Ronald Mason, Jr. (President), Dr. Felix Okojie (Provost and Vice President for Research and Federal Relations), and Dr. Mary Myles (Director of Title III Program) for their administrative support.

I would like to acknowledge the authors for their involvement and cooperation, and for their outstanding contributions to advancing scientific research and facilitating informed decision making in the critical area of environmental sustainability and public health protection. Special thanks are also extended to all the peer-reviewers who took time from their busy schedules to carefully and critically review each of the manuscripts. Special appreciations are also extended to all my colleagues and staff who worked very hard to make the symposium a total success.

## Figures and Tables

**Figure 1.**
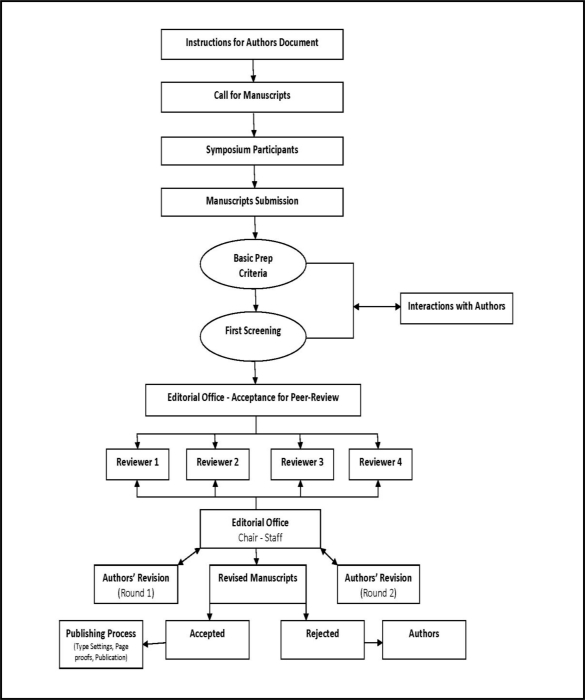
Manuscripts Peer-Review Process.

